# Bioprospecting the Rodriguan Lime (*Citrus aurantifolia* Swingle) as a Novel Source of Antioxidants and Antimicrobials for Food Application

**DOI:** 10.1155/ijfo/9985071

**Published:** 2025-05-24

**Authors:** Liza Cloete, Anton Venter, Mohammad Naushad Emmambux, Deena Ramful-Baboolall, Brinda Ramasawmy, Swaleha Hudaa Neetoo, Carene Picot-Allain, Kwaku Gyebi Duodu

**Affiliations:** ^1^Department of Agricultural and Food Science, Faculty of Agriculture, University of Mauritius, Reduit, Mauritius; ^2^Department of Consumer and Food Sciences, University of Pretoria, Hatfield, South Africa; ^3^Department of Agricultural Production and Systems, Faculty of Agriculture, University of Mauritius, Reduit, Mauritius; ^4^Biopharmaceutical Unit, Centre for Biomedical and Biomaterials Research, University of Mauritius, Reduit, Mauritius

**Keywords:** antimicrobial activity, antioxidant activity, citrus fruit peels, coumarins, flavonoids, liquid chromatography–mass spectrometry

## Abstract

In view of bioprospecting the Rodriguan lime (*Citrus aurantifolia* Swingle) as a novel antioxidant and antimicrobial for the food industry, its bioactivities were compared with those of the Mauritian pamplemousses (*Citrus maxima*) and the Rodriguan grapefruit (*Citrus × paradisi* Macfad). The Rodriguan lime, “Limon Rodrigues,” is also known as the Mexican lime (*Citrus aurantiifolia*, Swingle) or key lime. All citrus peel extracts tested in the study—namely, the Rodriguan lime, Mauritian pamplemousses, and Rodriguan grapefruit—exhibited comparable antioxidant activity in the ferric reducing antioxidant power (FRAP) (14.50 ± 3.11, 12.96 ± 0.97, and 14.77 ± 1.47) and CUPRAC (cupric reducing antioxidant capacity) (0.71 ± 0.20, 0.50 ± 0.04, and 0.59 ± 0.11) assays. The Rodriguan lime extract had the lowest overall minimum inhibitory concentration (MIC) of 5–10 mg/mL against *Staphylococcus aureus*, *Salmonella enterica*, *Listeria monocytogenes*, *Bacillus cereus*, and *Lactobacillus plantarum*. Although the Rodriguan grapefruit peel had the highest total phenolic content (64.53 ± 3.25 mg GAE/g extract) (*p* < 0.05), its total flavonoid content was not significantly different from that of the Rodriguan lime peel (*p* > 0.05). LC-MS data revealed that the Rodriguan grapefruit extract possessed the highest overall concentration of flavonoids (4821.1 mg RE/kg) and coumarins (13476 mg CE/kg), although the Rodriguan lime peel extract exhibited a relatively unique flavonoid and coumarin profile. Citrus flavonoids and coumarins exhibit diverse biological functions, including antidiabetic, antimicrobial, antifungal, hypotensive, antioxidant, carminative, antibacterial, larvicidal, antiviral, uricosuric, antiyeast, antihepatotoxic, and antimutagenic activities. Additionally, they demonstrate significant anticancer, cardiovascular-protective, and neuroprotective properties. These multifunctional bioactive compounds highlight the potential of citrus-derived substances in therapeutic and preventive health applications. Given its broad antimicrobial spectrum and diverse phytochemicals, the Rodriguan lime extract shows potential for applications in the functional food and nutraceutical industries.

## 1. Introduction

The genus *Citrus* spp., belonging to the family Rutaceae, is one of the most significant fruit crops globally. According to the Food and Agriculture Organization (FAO), global citrus production reached 143.8 million tons in 2019, with cultivation spanning over 140 countries, making citrus one of the most commercialized horticultural products [1]. Citrus fruits are categorized into sweet oranges, lemons/limes, mandarins (including clementines and tangerines), and grapefruits (including pummelos) [2]. Approximately 80% of citrus harvests are utilized in the juice industry, generating substantial by-products—comprising peels, seeds, and pulp—which represent up to 55% of the fruit's weight. These by-products pose significant environmental disposal challenges, underscoring the necessity of value-added utilization strategies to mitigate their ecological impact [3].

Citrus by-products are rich in dietary fiber, minerals, and bioactive compounds, including vitamins B, ascorbic acid, coumarins, carotenoids, flavonoids, and essential oils (terpenes and limonoids) [2, 4]. The peel, which constitutes 50% of the fruit's mass, is particularly abundant in phenolic compounds such as flavanones, flavonols, and polymethoxylated flavones, which are rare in other plant species [4]. Key flavonoids include hesperidin, naringin, narirutin, and eriocitrin [5, 6]. Additionally, coumarins and furanocoumarins, such as bergapten and isopimpinellin, have been identified in citrus peels, where they play roles in pathogen defense [7, 8]. These phytochemicals have been extensively studied for their physiological, ecological, and industrial applications, exhibiting a broad range of bioactivities including antioxidant, antimicrobial, antifungal, anti-inflammatory, and anticancer properties [6, 9, 10].

The Rodriguan lime (*Citrus aurantifolia* Swingle), also known as “Limon Rodrigues,” is the predominant citrus variety on Rodrigues, an island in the Republic of Mauritius. This species stands out due to its exceptional drought tolerance and ability to thrive in marginal growing conditions [11]. Unlike the common key lime, the Rodriguan lime has remained genetically isolated due to the island's geographical separation, resulting in unique phytochemical properties [12]. Additionally, it reaches full maturity while retaining its distinctive green rind, making it competitive with limes from other regions where early yellowing occurs due to climate. Despite its ecological and agricultural advantages, the phytochemical composition and bioactivities of the Rodriguan lime remain largely unexplored, representing a significant research gap.

This study is the first to characterize the Rodriguan lime peel extract and compare its bioactive properties with those of Rodriguan grapefruit (*Citrus × paradisi*) and Mauritian pamplemousses (*Citrus × maxima*). The novelty of this work lies in demonstrating the Rodriguan lime's unique phytochemical profile, exceptional drought tolerance, and novel antimicrobial properties. These features position the Rodriguan lime as an ideal candidate for value-added applications in functional foods and nutraceuticals. Furthermore, the study highlights its potential use in developing edible coatings enriched with natural antimicrobials and antioxidants. Such coatings could be applied to minimally processed and whole fresh fruits and vegetables, extending their shelf life while meeting the growing demand for clean-label, sustainable food preservation solutions. This approach aligns with global trends in reducing food waste, promoting sustainable agriculture, and enhancing food security.

## 2. Methodology

### 2.1. Collection of Fruits

**Figure 1:**
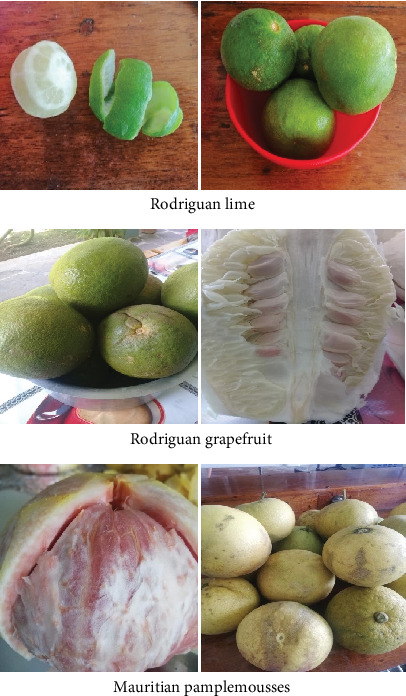
Pictures of the different citrus varieties.

The Rodriguan lime (*Citrus aurantifolia* Swingle) and Rodriguan grapefruit (*Citrus × paradisi* Macfad), which exhibited no signs of insect infestation or blemishes, were collected from Rodrigues Island, Mauritius (Figure 1). The Mauritian pamplemousses (*Citrus maxima*) was collected from Pamplemousses (20.1095° S, 57.5816° E), Mauritius, in 2021 and 2022.

### 2.2. Solvent Extraction and Preparation of Freeze-Dried Extracts

The fruits were washed with distilled water and peeled, and the peels were dried in an oven for 4 days at 65°C, then ground to a powder using a blender. A known quantity of each peel powder (2.5 g) was added into separate Erlenmeyer flasks containing 50 mL of 80% ethanol (v/v) and left to macerate for 48 h with occasional stirring [13]. The solvent was decanted, and fresh solvent was added to the residue, which was again left to macerate overnight. The extracts were pooled and filtered through sterile Whatman No. 1 filter paper and allowed to evaporate until dry. The condensed peel extracts were used for subsequent analysis (provide reference). Extractions were performed in triplicate.

Ethanol was removed from the crude peel extracts by rotary evaporation, and the crude extracts were then freeze-dried to remove excess moisture. The final freeze-dried crude extracts were in a hygroscopic powdered form and subsequently sealed in airtight containers and placed in frozen storage until needed for further analysis.

### 2.3. Characterisation of the Citrus Peel Extracts

#### 2.3.1. Total Phenolic Content

The Folin–Ciocalteu method, as described by Nickavar and Esbati [14], was used to determine the total phenolic content. The reaction mixture contained 2500 *μ*L of a 10-fold dilution of Folin–Ciocalteu reagent, 500 *μ*L of the sample, and 2000 *μ*L of sodium carbonate (7.5%). The reaction mixture was allowed to react for 30 min at room temperature. The sample's total phenolic content was then measured at 760 nm using a spectrophotometer (Jenway Spectrophotometer 7305). The total phenolic content was measured in milligrams of gallic acid equivalent (GAE) per gram of extract after being computed using a gallic acid standard curve.

#### 2.3.2. Total Flavonoid Content

The total flavonoid content was determined using the aluminum chloride colorimetric method described by Amaeze et al. [15]. Two milliliters of the sample and 2 mL of the 2% aluminum chloride solution made up the reaction mixture. After 30 min of reaction time at room temperature, the mixture's absorbance was measured at 420 nm. Using a rutin standard curve, the total flavonoid content was calculated. The results were reported as milligrams of rutin equivalents (RE) per gram of extract.

#### 2.3.3. Ferric Reducing Antioxidant Power (FRAP) Assay

The FRAP assay, as described by Benzie and Strain [16], was used to determine the samples' ferric reducing antioxidant ability, with Trolox used as a standard. A total of 2850 *μ*L of FRAP solution (25 mL hydrated ferric chloride solution [20 mM], 2.5 mL 2,4,6-tripyridyl-s-triazine [10 mM in 40 mM HCl], and acetate buffer [300 mM and pH 3.6]) were combined with the sample. After 30 min of dark incubation, the reaction mixture was measured for absorbance at 593 nm using a spectrophotometer (Jenway Spectrophotometer 7305). The results were reported as milligrams of Trolox equivalents (TE) per gram of crude extract.

#### 2.3.4. Nitric Oxide (NO) Radical Scavenging Assay

By utilizing 0.1% w/v naphthylethylenedihydrochloride, NO was generated from sodium nitroprusside and quantified using the Griess reagent. A total of 50 *μ*L of sodium nitroprusside (10 mM) and 50 *μ*L of sample were added to the reaction mixture, which was then incubated at room temperature for 180 min. Following the incubation period, the mixture was mixed with 125 *μ*L of Griess reagent and incubated for 10 min. Using a spectrophotometer (Jenway Spectrophotometer 7305), the absorbance was measured at 546 nm. The formula for calculating the inhibition percentage was %inhibition = [(negative control sample Abs)/negative control Abs] × 100. The IC50 was then determined using the % inhibition value.

#### 2.3.5. Xanthine Oxidase (XO) Inhibition

In summary, 2.9 mL of phosphate buffer (pH 7.4), 0.1 mL of enzyme solution (0.04 units/mL), and 1 mL of the sample were combined, and the mixture was allowed to incubate at room temperature for 15 min. After adding 2 mL of xanthine solution (10 mM) and allowing the mixture to incubate at room temperature for 30 min, the reaction was initiated. One milliliter of HCl (1 N) was added to terminate the reaction, and a UV spectrophotometer (Jenway Spectrophotometer 7305) was used to detect absorbance at 290 nm.

#### 2.3.6. Cupric Reducing Antioxidant Capacity (CUPRAC)

The method described by Grochowski et al. [17] was used to determine the CUPRAC of the samples. The peel extract (0.5 mL) was mixed with a reaction mixture containing CuCl2 (1 mL and 10 mM), neocuproine (1 mL and 7.5 mM), and NH4CH3CO2 buffer (1 mL, 1 M, and pH 7.0) and incubated at room temperature. The absorbance was measured at 450 nm using a spectrophotometer (Jenway Spectrophotometer 7305) after 30 min, and the results were expressed as milligrams TE per gram extract.

#### 2.3.7. Identification and Quantification of Coumarins and Flavonoids Using Liquid Chromatography–Mass Spectrometry (LC-MS)

Phenolic compounds were analyzed by chromatographic analysis with a quenched ion mobility system as described by Stander et al. [18]. For coumarins, analysis was performed according to Dugo et al. [19]. Determination was carried out by comparing the chromatograms and retention times of the phenolic components in the extract with external standards of phenolic acids and flavonoids. Additionally, MS/MS fragmentation data and UV spectra were compared with those of phenolic compounds reported in the literature. Quantification was done by comparing integrated peak areas of phenolic compounds and coumarins in extracts with those of standards, namely, rutin and coumarin.

#### 2.3.8. Determination of Antimicrobial Activity of the Citrus Extracts

Foodborne pathogens *Staphylococcus aureus* (ATCC 25923), *Bacillus cereus* (ATCC 14579), *Salmonella enterica* sv Typhimurium (ATCC 14028), *Listeria monocytogenes* (ATCC 13932), and *Escherichia coli* (ATCC 25922) and spoilage organisms *Lactobacillus* sp. (ATCC 15578), *Issatchenkia orientalis* (ATCC 6258), *Penicillium citrinum* (ATCC 9849), and *Aspergillus niger* (ATCC 1015) were used as test microorganisms. Cultures of bacteria were grown for 24 h in 10 mL of nutrient broth (HiMedia) at 37°C and were maintained on differential HiCrome agar (HiMedia) at 4°C.

##### 2.3.8.1. Determination of Antimicrobial Effect Using Disc Diffusion Assay

The assessment of the antibacterial activity of natural extracts using the disc diffusion assay was conducted according to the standard method described by Bauer et al. [20]. The positive control used was chloramphenicol, a broad-spectrum antibiotic, prepared by dissolving 50 mg of chloramphenicol powder (HiMedia, India) in 60 *μ*L of ethanol and 4.94 mL of distilled water to achieve a final concentration of 10 mg/mL. For the positive control, the different crude extracts were dissolved in 99% ethanol and distilled water to achieve a final concentration of 100 mg/mL. The negative or solvent control was prepared by mixing 60 *μ*L of 99% ethanol (Supplies Solutions Ltd) with 4.94 mL of distilled water. Culti-Loops for each foodborne pathogen and spoilage organism were cultured on differential HiCrome media (HiMedia) and incubated for 24 h at 37°C. After incubation, a loopful of the colonies was transferred to 10 mL of nutrient broth (HiMedia) and incubated for another 24 h at 37°C. After 24 h, 1 mL of the broth was transferred to 9 mL of fresh nutrient broth for incubation at 37°C for another 24 h to ensure that the culture had reached the late-log phase. The resulting broth was serially diluted in 0.1% sterile buffered peptone water (HiMedia) to obtain a bacterial culture with a final density of 5 log CFU/mL. An inoculum of 0.1 mL of each culture was then spread-plated on the respective media. On each plate, three discs were placed equidistant from one another using sterile tweezers. An aliquot of 100 *μ*L of the different solutions, namely, the positive control (chloramphenicol), the negative control (99% ethanol), and the extract solutions, was pipetted onto each disc. Depending on the bacterial strain used, the plate was incubated for 18–24 h at 37°C. The inhibition zones were examined after incubation using a ruler. To ensure reliability, the test was repeated three times [21].

##### 2.3.8.2. Determination of Antimicrobial Effects Using the Minimum Inhibitory Concentration (MIC) Assay

The microdilution method was used to determine the MICs as adapted from Eloff [22]. The different bacterial strains were grown in liquid media as described above. After incubation, a loopful of the colonies was transferred to 10 mL of nutrient broth (HiMedia) and incubated for another 24 h at 37°C. After 24 h, 1 mL of the broth was transferred to 9 mL of fresh nutrient broth for incubation at 37°C for another 24 h to ensure that the culture had reached the late-log phase. The resulting broth was serially diluted in 0.1% sterile buffered peptone water (HiMedia) to obtain a bacterial culture with a final density of 5 log CFU/mL, which was used for the antibacterial screening assay [23].

Crude citrus extract suspensions of different concentrations (5, 10, 25, 50, and 100 mg/mL) were prepared for antimicrobial analysis by dissolving the extracts in distilled water. Aliquots of 100 *μ*L of the crude citrus suspensions of different concentrations were added to the wells of the plates, followed by 100 *μ*L of standardized inoculum (5 log CFU/mL) of the tested microorganisms. The plates were then incubated at 37°C for 24 h. An aliquot of 40 *μ*L of 0.3 mg/mL *p*-iodonitrotetrazolium (INT) was then added to each well and incubated for about 30 min. The MIC was defined as the lowest concentration at which microbial growth was inhibited. A completely clear well indicated inhibition of growth, while a pink colour indicated growth in the wells. Distilled autoclaved water was used as the negative (solvent) control. For the positive control, 200 *μ*L of the respective organisms was added to the last well of each replicate. The experiment was carried out in triplicate.

#### 2.3.9. Data and Statistical Analysis

All experiments were performed in at least two independent replicates. LC-MS data were analyzed using MassLynx software. TPC, TFC, FRAP, CUPRAC, NO radical scavenging assay, XO inhibition, and microbial population density data were statistically analyzed using a single-factor ANOVA followed by Tukey's post hoc test using Minitab Release 17. Statistical significance was assigned to *p* values less than 0.05 according to the procedure of Snedecor and Cochran [24].

## 3. Results and Discussion

### 3.1. Total Phenolic Content, Total Flavonoid Content, and Antioxidant Properties of Citrus Peel Extracts

The Rodriguan grapefruit peel showed the highest total phenolic content (64.53 ± 3.25 mg GAE/g extract) (*p* < 0.05) (Table 1). Elkhatim et al. [25] also found that grapefruit peels had the highest total phenolic content, followed by lemons and oranges (Table 1). Further, Li et al. [26] reported that grapefruit peels have higher total phenolic content than mandarins, lemons, and orange peels [27]. The total flavonoid content of the Rodriguan lime peel (53.48 ± 5.77 mg RE/g) and the Rodriguan grapefruit peel (49.34 ± 1.61 mg RE/g) was higher than that of the Mauritian pamplemousses (36.09 ± 0.35 mg RE/g) (*p* < 0.05).

The scavenging of radicals by antioxidants most commonly occurs via two mechanisms: the transfer of either a hydrogen atom or an electron to convert the radical into a stable compound [28]. Antioxidants neutralize radicals primarily through two key mechanisms: hydrogen atom transfer (HAT) and single-electron transfer (SET), both of which stabilize reactive species and prevent oxidative damage [28]. The FRAP assay evaluates an extract's electron-donating capacity by measuring the reduction of Fe^3+^ to Fe^2+^ in an acidic environment, reflecting its total reducing power. Similarly, the CUPRAC assay assesses the reduction of Cu^2+^ to Cu^+^ in a near-neutral pH, making it more physiologically relevant and capable of detecting a broader range of antioxidants [29]. Both the CPURAC and FRAP assays functions through SET. The results of the FRAP and CUPRAC assays (Table 2) did not show significant differences (*p* > 0.05) among the three citrus extracts, suggesting comparable overall reducing power across the samples.

The scavenging of NO radicals by antioxidants typically involves HAT. The ability of the extracts to scavenge radicals varied, as reflected in the NO radical scavenging assay. Mauritian pamplemousses (0.18 ± 0.02) and Rodriguan grapefruit (0.18 ± 0.02) extracts exhibited significantly (*p* < 0.05) lower IC50 values than the Rodriguan lime (0.22 ± 0.01) in the NO radical scavenging assay. The lower NO scavenging activity of Mauritian pamplemousses and Rodriguan grapefruit compared to Rodriguan lime is likely attributed to their higher total flavonoid contents. Erba et al. [30] found a statistically significant and strong linear correlation (*r* = 0.975, *p* = 0.025) between total flavonoid content and DPPH, suggesting that antioxidant activity depends on the concentration of flavonoids present. XO primarily functions through SET [31]. Data gathered from the present study revealed that only the Mauritian pamplemousses peel extract inhibited XO activity (Table 2).

Phenolic and flavonoid compounds are produced by plants to protect themselves or promote their growth in unfavorable conditions. However, they are also reported to play an important role against oxidative stress and damage due to their radical neutralization, iron binding, and reducing capacity [32]. Phenolic and flavonoid molecules are important antioxidant components responsible for quenching free radicals based on their ability to provide hydrogen atoms [33]. They also have ideal structural characteristics for scavenging free radicals [34, 35].

### 3.2. In Vitro Antimicrobial Efficacy of the Citrus Peel Extracts

**Figure 2:**
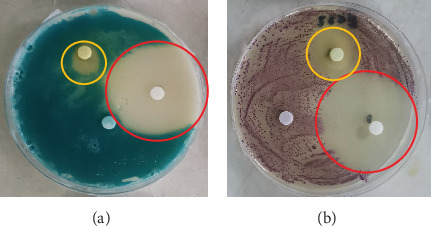
(a) Inhibition zones of Rodriguan lime extract (yellow circle) and the positive control (chloramphenicol) (red circle) against *S. aureus*. (b) Inhibition zones of Rodriguan lime extract (yellow circle) and the positive control (chloramphenicol) (red circle) against *E. coli.*

The disc diffusion assay was performed on a variety of spoilage organisms using the different citrus extracts. The Rodriguan grapefruit extract showed no inhibitory effect against *S. aureus*, *Salmonella*, *E. coli*, *Listeria*, *Bacillus*, or *Penicillium* (Table 3). The Mauritian pamplemousses extract showed inhibitory effects against *Salmonella*, *E. coli*, and *Listeria*, as shown in Table 3. Literature reports indicate that extracts from citrus peels have antimicrobial activity against a variety of microorganisms, supporting our findings [36]. The Rodriguan lime extract showed inhibitory effects against the following organisms: *S. aureus* (1.5 ± 0.36 cm), *S. enterica* (1.8 ± 0.53 cm), *E. coli* (2.1 ± 0.46 cm), *L. monocytogenes* (1.8 ± 0.7 cm), *B. cereus* (1.6 ± 0.69 cm), and *Lactobacillus plantarum* (1.3 ± 0.12 cm) (Table 3) (Figure 2). Thus, the Rodriguan lime extract showed the broadest efficacy against the tested foodborne organisms compared to the Mauritian pamplemousses and Rodriguan grapefruit extracts, which showed little to no inhibition according to the disc diffusion assay.

#### 3.2.1. In Vitro Antimicrobial Assay for Different Citrus Extracts Against Several Spoilage Organisms Using Disc Diffusion Assay

The disc diffusion assay is widely known to be inaccurate since the inhibition zones need to be visually assessed with the naked eye [37–39]. On the other hand, the MIC assay is a much better technique to assess the effect of these extracts against the tested organisms. Furthermore, high concentrations (100 mg/mL) of the different extracts were needed to indicate clear inhibition zones around the inoculated discs (Figure 2), given the low sensitivity of the assay [40]. The MIC assay was subsequently used to determine more precisely the concentration of extracts effective at inhibiting microbial growth, given its greater reliability and consistency [40].

#### 3.2.2. In Vitro Antimicrobial Assay for Different Citrus Extracts Against Several Spoilage Organisms Using the MIC Assay

Table 4 shows that the Rodriguan lime extract consistently had the lowest overall MIC, in the range of 5–10 mg/mL, against all the tested organisms except for *E. coli*, where the MIC was 25 mg/mL. The Rodriguan grapefruit extract and Mauritian pamplemousses extract had generally higher overall MICs of 10 and 25 mg/mL, respectively, against their tested organisms compared to the Rodriguan lime extract (5–10 mg/mL). According to Chaisiwamongkhol et al. [41], key lime extracts have been shown to inhibit a range of spoilage organisms, thereby extending the shelf life of perishable food products. They are also effective against foodborne pathogens such as *E. coli*, *Salmonella* spp., *L. monocytogenes*, and *Staphylococcus aureus*, which are responsible for serious foodborne illnesses. This dual action highlights the potential of key lime extracts as natural preservatives in the food industry, offering a safer and more sustainable alternative to synthetic chemical additives [41].

### 3.3. Identification and Quantification of Coumarins and Flavonoids in the Citrus Peel Extracts

Table 5 shows the chromatographic and mass spectral data of coumarins and flavonoids identified in extracts from the peels of the three citrus varieties.

#### 3.3.1. Flavonoid Derivatives Identified

##### 3.3.1.1. Kaempferol Derivatives

Peak 1, with a retention time of 2.29 min and a maximum absorption wavelength (*λ*_max_) of 267 and 351 nm, with the main fragment of m/z 433.113, was identified as kaempferol-7-*O*-alpha-l-rhamnoside. This identification was based on the kaempferol aglycone (m/z 287.055), corresponding to the loss of a rhamnose (−146 amu) moiety.

##### 3.3.1.2. Apigenin Derivatives

All seven apigenin derivatives identified produced a fragment at m/z 271.090, corresponding to the apigenin molecule. Peaks 3, 5, and 24 were identified as apigenin-7-*O*-neohesperidoside, its isomer, and apigenin-rutinoside, respectively, based on the loss of a rutinose (−308 amu). Peak 10 was identified as apigenin 7-(6″-malonyl neohesperidoside) due to the loss of a malonyl hexose (−248 amu) and a rhamnosyl (−146 amu) moiety [−394 amu] [42]. Peak 18 was identified as apigenin 7-*O*-(2G-rhamnosyl) gentiobioside based on the loss of a glucose (−162 amu) unit, a rutinose (−308 amu), and the consecutive loss of a glucose (−162 amu) molecule. Peaks 26 and 27 were identified as Camellianin A (apigenin 7-*O*-glucoside 4′-acetate) and vitexin 2″-*O*-rhamnoside 6″-acetate, respectively. For both compounds, fragmentation resulted in the loss of a rhamnoside (−146 amu) and an acetyl hexose (−204 amu) fragment [42].

##### 3.3.1.3. Naringenin Derivatives

Peak 2, with a retention time of 2.76 min, a maximum absorption wavelength (*λ*_max_) of 282 and 327 nm, and a main fragment at m/z 273.077, was tentatively identified as naringenin. Fragmentation produced fragments at m/z 153.02, corresponding to the 1,3A fragment from the retro-Diels–Alder (RDA) cleavage of the naringenin aglycone at bond Positions 1 and 3 in the C ring [43, 44]. Peak 9 was identified as 6″-*O*-malonyl naringin based on the loss of a malonyl hexose (−204 amu), followed by the loss of a hexose (−162 amu) moiety [42]. Peak 6, with a retention time of 3.07 min, a maximum absorption wavelength (*λ*_max_) of 284 nm, and a main fragment of m/z 609.182, was identified as diosmin (diosmetin 7-*O*-rutinoside). Fragmentation produced an m/z 301.070 fragment, corresponding to the loss of a rutinose (−308 amu) fragment [42]. Peak 28, with a retention time of 24.66 min and a maximum absorption wavelength (*λ*_max_) of 321 nm, was identified as Icariside II based on the main fragment of m/z 515.192.

#### 3.3.2. Coumarin Derivatives Identified

##### 3.3.2.1. Meranzin Derivatives

All three meranzin derivatives were identified based on the main fragment of m/z 261.113. Peaks 7, 11, 16, and 17 were identified as meranzin hydrate, auraptenol, meranzin, and isomeranzin, respectively. Peak 13, with a retention time of 4.92 min, a maximum absorption wavelength (*λ*_max_) of 322 nm, and a parent fragment of m/z 177.055, was identified as 7-methoxycoumarin based on the consecutive loss of two carbon monoxide molecules [−56 amu], producing an m/z 121.06 fragment. The m/z 147.045 fragment corresponds to the loss of a methoxy group (−30 amu) [45]. Peak 14 was identified as limettin (citropten) based on the main fragment at m/z 207.065. Further fragmentation yielded ionic fragments of m/z 192.042 and 149.023. The m/z 192.042 fragment resulted from the loss of a methyl (−15 amu) molecule, while the m/z 149.023 fragment was due to the loss of a methyl (−15 amu) followed by the loss of a C_2_H_3_O (−43 amu) moiety [−58 amu] [45].

##### 3.3.2.2. Furanocoumarin Derivatives

Peak 19, with a retention time of 4.62 min, a maximum absorption wavelength (*λ*_max_) of 351 nm, and a parent fragment at m/z 287.092, was identified as oxypeucedanin based on the m/z 203.034, which is the hydroxy psoralen corresponding to the loss of a trimethyl epoxide (−84 amu) moiety [46]. Peak 15 was identified as isopimpinellin based on the parent fragment at m/z 247.060 and the main ionic fragment at m/z 217.013, corresponding to the consecutive loss of two methyl [−30 amu] groups [47]. Peak 18 was identified as an umbelliferone derivative based on the main fragment at m/z 163.039. Peak 20 was identified as a bergamottin derivative based on the main ionic fragment produced as a bergaptol ion at m/z 203.034 [3]. Peak 21, with a retention time of 17.75 min, a maximum absorption wavelength (*λ*_max_) of 271 and 312 nm, and a parent fragment at m/z 369.170, was identified as 5-geranyloxy-8-methoxypsoralen. Fragmentation produced ions at m/z 233.045, corresponding to the loss of a geranyl group (−136 amu) [48]. Peak 22 was identified as bergamottin based on the molecular formula C_11_H_7_O_4_, with a parent ion at m/z 339.160. Fragmentation produced a bergaptol ion at m/z 203.034 [3]. Peak 23 was identified as 7-geranyloxy-6-methoxycoumarin based on a main parent fragment at m/z 329.175 and the loss of a geranyl (−136 amu) moiety, resulting in an m/z 193.050 fragment [48]. Peak 25 was identified as coumestrol-rutinoside based on the coumestrol main fragment with m/z 267.046, corresponding to the loss of a rutinoside (−308 amu) moiety [42].

The Rodriguan grapefruit extract possessed the largest overall concentration of flavonoids (4821.1 mg RE/kg), followed by the Mauritian pamplemousses (3178.6 mg RE/kg) and the Rodriguan lime (1076.0 mg RE/kg) (Table 6). The major flavonoids identified in the Rodriguan grapefruit extract were apigenin-rutinoside (1481.7 mg RE/kg), naringenin (1200.1 mg RE/kg), and diosmin (diosmetin 7-*O*-rutinoside) (828.0 mg RE/kg). The major flavonoids identified in the Mauritian pamplemousses extract sample were apigenin-rutinoside (1221.0 mg RE/kg), naringenin (947.8 mg RE/kg), and vitexin 2″-*O*-rhamnoside 6″-acetate (449.0 mg RE/kg). The major flavonoids identified in the Rodriguan lime extract sample were hesperidin (661.6 mg RE/kg), Icariside II (393.81 mg RE/kg), and kaempferol-7-*O*-alpha-l-rhamnoside (11.54 mg RE/kg).

From Table 6, two specific flavonoids were identified in the Rodriguan lime extract but not in the Rodriguan grapefruit or Mauritian pamplemousses samples, that is, hesperidin and kaempferol-7-*O*-alpha-L-rhamnoside. Hesperidin is a flavanone glycoside present in citrus peel. Akbar et al. [5] found that hesperidin exhibited notable antimicrobial, anti-inflammatory, antioxidant, and antitumor activities. Similarly, Iranshahi et al. [6] also found that hesperidin has antibacterial activity and suggested that these properties may be due to mechanisms such as the activation of the host immune system, disruption of bacterial membranes, and interference with bacterial enzymes [49]. In addition to its anticancer and anti-inflammatory effects, kaempferol and its related compounds also have antibacterial, antifungal, and antiprotozoal activities [50].

The Rodriguan grapefruit extract possessed the largest overall concentration of coumarins (13,476 mg CE/kg), followed by the Mauritian pamplemousses extract (Table 6). The major coumarins identified in the Rodriguan grapefruit extract sample were meranzin (5317.3 mg CE/kg), auraptenol (4853.8 mg CE/kg), and isomeranzin (2129.6 mg CE/kg). The major coumarins identified in the Mauritian pamplemousses sample were meranzin (3287.4 mg CE/kg), auraptenol (2900.6 mg CE/kg), and isomeranzin (827.4 mg CE/kg). The major coumarins identified in the Rodriguan lime extract sample were isopimpinellin (1731.5 mg CE/kg), 7-geranyloxy-6-methoxycoumarin (1616.5 mg CE/kg), and limettin (1372.0 mg CE/kg).

Looking at Table 7, a number of coumarins were identified in the Rodriguan lime extract but were not present in the other two peel extracts, that is, 9-hydroxy-4-methoxypsoralen 9-glucoside, 7-methoxycoumarin, limettin, isopimpinellin, 5-geranyloxy-8-methoxypsoralen, and 7-geranyloxy-6-methoxycoumarin. According to findings by Piao et al. [51], 9-hydroxy-4-methoxypsoralen displayed powerful antioxidant effects against the DPPH radical and against renal epithelial cell injury. Their results also showed that the aromatic hydroxy group plays a significant antioxidant role by stabilizing the radical form and participating in electron delocalization.

In fact, 7-methoxycoumarin has been recently identified by Han et al. [52], who also reported its strong antibacterial activity against *Ralstonia solanacearum*, a Gram-negative organism. This is congruent with our finding that Rodriguan lime extract also showed inhibitory activity against Gram-negative bacteria *Salmonella* and *E. coli.* 7-Methoxycoumarin has also been demonstrated to restrict biofilm formation, induce bacterial cell membrane lysis, and significantly suppress virulence-associated genes. Additionally, Yang et al. [53] found that 7-methoxycoumarin, which contains a methoxy functional group on the coumarin skeleton, has notable antibacterial activity against foodborne pathogens. In addition to 7-methoxycoumarin, isopimpinellin was another novel molecule identified within the Rodriguan lime. This molecule was reported to exhibit antimicrobial activity against *Cryptococcus neoformans* and *Mycobacterium intracellulare* [54]. Furthermore, Madeiro et al. [7] found that isopimpinellin potentiated the effect of erythromycin, which could be attributed to its mechanism of action as an efflux pump inhibitor of bacteria. Little information is available on the scientific application of 7-geranyloxy-6-methoxycoumarin. However, as a coumarin derivative, a type of compound found in many plants, it has been studied for its potential medicinal properties (e.g., antioxidant, anti-inflammatory, antitumor, and antimicrobial activities). Furthermore, 7-geranyloxy-6-methoxycoumarin has been found to have potential therapeutic applications in a variety of diseases, including cancer, diabetes, and Alzheimer's [55, 56]. The antifungal activity of Rodriguan lime against *Penicillium citrinum* and *Aspergillus niger* could likely be attributed to the molecule limettin. In fact, Ramírez-Pelayo et al. [8] found that limettin exhibited inhibitory effects against the fungus *Colletotrichum* sp., surpassing those of known phytoalexins scoparone and umbelliferone.

Flavonoids and coumarins exhibit antimicrobial properties through diverse mechanisms, making them effective against both Gram-positive and Gram-negative bacteria. Flavonoids disrupt bacterial membranes by increasing their permeability, leading to the leakage of intracellular contents, as seen with compounds like quercetin and catechins [57]. They also inhibit essential bacterial enzymes, such as DNA gyrase and *β*-lactamase, which are critical for replication and survival—examples include apigenin and luteolin [58]. Additionally, flavonoids chelate vital metal ions such as iron, depriving bacteria of necessary resources for metabolic functions [59]. Furthermore, flavonoids interfere with biofilm formation, either preventing its development or disrupting existing biofilms, a key bacterial defense mechanism [57].

Since the overall concentration of coumarins and flavonoids was not the highest within the Rodriguan lime extract sample (Tables 6 and 7), its antimicrobial efficacy could likely be attributed to other specific compounds identified within the sample or the synergistic effect of coumarins and flavonoids found within the extract.

### 3.4. Conclusion

Rodriguan lime was found to exhibit antioxidant activity comparable to that of Mauritian pamplemousses and Rodriguan grapefruit. However, the Rodriguan lime extract exhibited a lower MIC (10 mg/mL) against several spoilage organisms compared to the other extracts tested (25 mg/mL). This could be attributed to the compounds uniquely identified within the Rodriguan lime sample. Rodriguan lime extract was found to contain several compounds not present in the Mauritian pamplemousses and Rodriguan grapefruit extracts, namely, 9-hydroxy-4-methoxypsoralen 9-glucoside, 7-methoxycoumarin, limettin, isopimpinellin, 5-geranyloxy-8-methoxypsoralen, and 7-geranyloxy-6-methoxycoumarin. Citrus peels, rich in valuable bioactive compounds like phenolics and coumarins, could serve as active ingredients in food products or even replace synthetic preservatives entirely [9]. Incorporating these bioactive compounds into an antimicrobial edible coating offers significant potential to extend shelf life, improve food safety, and minimize dependence on synthetic preservatives. Natural bioactive compounds can also be used as potential therapeutic agents that may eventually replace antibiotics that are no longer effective against bacterial pathogens.

## Figures and Tables

**Table 1:** Percentage yield, total phenolic, and flavonoid content of the citrus peel extracts.

	**Yield % dry weight**	**Total phenolic content (mg gallic acid equivalents/g extract)**	**Total flavonoid content (mg rutin equivalents/g extract)**
Rodriguan lime	19	37.17 ± 5.07^b^	53.48 ± 5.77^a^
Mauritian pamplemousses	27	42.80 ± 0.60^b^	36.09 ± 0.35^b^
Rodriguan grapefruit	21	64.53 ± 3.25^a^	49.34 ± 1.61^a^

*Note:* Values are reported as mean ± standard deviation for three independent experiments. Different superscripts in each column represent significant difference between samples (*p* < 0.05).

**Table 2:** In vitro antioxidant activity of the citrus peel extracts.

	**Ferric reducing antioxidant power (mg TE/g extract)**	**Cupric ion reducing capacity (mg TE/g extract)**	**Nitric oxide radical scavenging (IC**_**50**_ **mg/mL)**	**Xanthine oxidase inhibition (IC**_**50**_ ** mg/mL)**
Rodriguan lime	14.50 ± 3.11^a^	0.71 ± 0.20^a^	0.22 ± 0.01^a^	-
Mauritian pamplemousses	12.96 ± 0.97^a^	0.50 ± 0.04^a^	0.18 ± 0.02^b^	0.13 ± 0.00
Rodriguan grapefruit	14.77 ± 1.47^a^	0.59 ± 0.11^a^	0.18 ± 0.02^b^	-

*Note:* Values are reported as mean ± standard deviation for three experiments; “-” indicates no activity. Different superscripts in each column represent significant difference between the tested samples (*p* < 0.05).

Abbreviation: TE: Trolox equivalents.

**Table 3:** Inhibition zones (centimeter) obtained for different citrus extracts against common pathogenic and spoilage organisms.

**Microorganisms**	**Solvent: dH**_**2**_ **O (negative control)**	**Antibiotic: Chloramphenicol (positive control)**	**Rodriguan lime**	**Rodriguan grapefruit**	**Mauritian Pamplemousses**
*Staphylococcus aureus*	0.0 ± 0.00^c^	4.6 ± 0.42^a^	1.5 ± 0.36^b^	0.0 ± 0.00^c^	0 ± 0.00^c^
*Salmonella enterica*	0.0 ± 0.00^c^	4.1 ± 0.17^a^	1.8 ± 0.53^b^	0.0 ± 0.00^c^	1.1 ± 0.1^b^
*Escherichia coli*	0.0 ± 0.00^c^	4.6 ± 0.58^a^	2.1 ± 0.46^b^	0.0 ± 0.00^c^	1.4 ± 0.17^b^
*Listeria monocytogenes*	0.0 ± 0.00^c^	4.7 ± 0.84^a^	1.8 ± 0.70^b^	0.0 ± 0.00^c^	0.8 ± 0.45^c^
*Bacillus cereus*	0.0 ± 0.00^c^	4.7 ± 0.93^a^	1.6 ± 0.69^b^	0.0 ± 0.00^c^	0.0 ± 0.00^c^
*Lactobacillus plantarum*	0.0 ± 0.00^c^	4.2 ± 0.61^a^	1.3 ± 0.12^b^	1.4 ± 0.85^c^	0.0 ± 0.00^c^
*Penicillium citrinum*	0.0 ± 0.00^c^	1.7 ± 0.06^b^	0.0 ± 0.00^c^	0.0 ± 0.00^c^	0.0 ± 0.00^c^

*Note:* Values are reported as mean ± standard deviation for three independent trials. Different superscripts between rows represent significant difference between the tested samples (*p* < 0.05). Different superscripts in each column represent significant difference between the organisms tested against (*p* < 0.05). (+) control: chloramphenicol. (−) control: distilled H_2_O.

**Table 4:** Minimum inhibitory concentration (MIC) (milligrams per milliliter) of the different citrus extracts against test organisms.

**Organisms**	**MIC (mg/mL)**		
	**Rodriguan grapefruit**	**Mauritian Pamplemousses**	**Rodriguan lime**
*Staphylococcus aureus*	25 ± 0.0	25 ± 0.0	10 ± 0.0
*Salmonella enterica*	25 ± 0.0	10 ± 0.0	10 ± 0.0
*Escherichia coli*	25 ± 0.0	25 ± 0.0	25 ± 0.0
*Listeria monocytogenes*	25 ± 0.0	25 ± 0.0	10 ± 0.0
*Bacillus cereus*	10 ± 0.0	10 ± 0.0	5 ± 0.0
*Lactobacillus plantarum*	25 ± 0.0	25 ± 0.0	10 ± 0.0
*Penicillium citrinum*	10 ± 0.0	10 ± 0.0	5 ± 0.0
*Aspergillus niger*	25 ± 0.0	25 ± 0.0	10 ± 0.0
*Issatchenkia orientalis*	25 ± 0.0	25 ± 0.0	10 ± 0.0

*Note:* Values are reported as mean ± STD for three independent replicates. (+) control: chloramphenicol. (−) control: distilled H_2_O.

**Table 5:** Retention time (*t*_R_), UV-visible absorption maxima (*λ*_max_), and mass spectral characteristics of coumarins and flavonoids found in Rodriguan grapefruit, Rodriguan lime, and Mauritian pamplemousses.

**t**_**R**_ ** (min)**	**λ**_max_ ** (nm)**	**Molecular formula (M** ^**+**^ **)**	**[M** ^**+**^ **] (m/z)**	**MS/MS (% intensity)**	**Proposed compound**	**Peak no.**
Flavonoid derivatives						
2.29	267, 351	C_21_H_21_O_10_	433.113	287.055 (100)	Kaempferol-7-*O*-alpha-l-rhamnoside	1
2.76	282, 327	C_15_H_13_O_5_	273.077	273.077 (100), 153.02 (21)	Naringenin	2
2.83	267, 335	C_27_H_31_O_14_	579.171	271.060 (100)	Apigenin 7-*O*-neohesperidoside	3
3.02	285	C_28_H_35_O_15_	611.197	303.089 (100), 153.018 (21)	Hesperidin	4
3.02	267, 335	C_27_H_31_O_14_	579.171	271.060 (100)	Apigenin 7-*O*-neohesperidoside isomer	5
3.07	284	C_28_H_33_O_15_	609.182	301.070 (100)	Diosmin (diosmetin 7-*O*-rutinoside)	6
3.45	282, 327	C_30_H_35_O_17_	667.187	273.076 (100), 463.157 (33)	6″-*O*-Malonyl naringin	9
4.04	332, 269	C_30_H_33_O_17_	665.170	271.060 (100)	Apigenin 7-(6″-malonyl neohesperidoside)	10
15.7	285	C_33_H_41_O_19_	741.22	579.171 (100), 271.06 (41)	Apigenin 7-*O*-(2G-rhamnosyl)gentiobioside	18
19.94	283	C_27_H_31_O_14_	579.174	579.174 (100), 271.060 (19), 151.00 (18)	Apigenin-rutinoside	24
21.72	280	C_29_H_33_O_15_	621.182	271.060 (58), 151.002 (70)	Camellianin A (apigenin 7-*O*-glucoside 4′-acetate)	26
22.24	283	C_29_H_33_O_15_	621.183	621.183 (100), 151.001 (40), 271.062 (30)	Vitexin 2″-*O*-rhamnoside 6″-acetate	27
24.66	321	C_27_H_31_O_10_	515.192	515.192 (100)	Icariside II	28

Coumarin derivatives						
3.41	282, 327	C_15_H_17_O_4_	261.113	189.055 (100), 243.103 (25), 131.05 (23), 103.057 (6)	Meranzin hydrate	7
3.56	312	C_18_H_19_O_10_	395.09	147.04 (100)	9-Hydroxy-4-methoxypsoralen 9-glucoside	8
4.58	323, 257	C_15_H_17_O_4_	261.114	189.055 (100), 243.103 (25), 131.05 (23), 103.057 (6)	Auraptenol	11
4.62	351	C_16_H_15_O_5_	287.092	203.034 (100)	Oxypeucedanin isomer	12
4.92	322	C_10_H_9_O_3_	177.055	121.06 (83), 147.045 (14)	7-Methoxycoumarin	13
6.81	330	C_11_H_11_O_4_	207.065	207.065 (100), 192.042 (50), 149.023 (41)	Limettin	14
7.17	267, 312	C_13_H_11_O_5_	247.060	217.013 (100)	Isopimpinellin	15
7.6	282, 329	C_15_H_17_O_4_	261.115	189.055 (100), 243.103 (25), 131.05 (23), 103.057 (6)	Meranzin	16
7.82	282, 330	C_15_H_17_O_7_	261.116	189.055 (100), 243.103 (25), 131.05 (23), 103.057 (6)	Isomeranzin	17
8.10	322	C_21_H_23_O_5_	355.154	355.154 (100), 163.039 (45), 189.055 (29), 261.111 (20)	Umbelliferone derivative	18
8.92	351	C_16_H_15_O_5_	287.092	203.034 (100)	Oxypeucedanin	19
13.58	310	C_23_H_21_O_5_	377.139	203.034 (100), 147.045 (22)	Bergamottin derivative	20
17.75	271, 312	C_22_H_25_O_5_	369.170	233.045 (100)	5-Geranyloxy-8-methoxypsoralen	21
18.03	309	C_11_H_7_O_4_	339.160	203.034 (100)	Bergamottin	22
18.29	323	C_20_H_25_O_4_	329.175	329.175 (100), 193.050 (23)	7-Geranyloxy-6-methoxycoumarin	23
20.25	337, 267	C_27_H_29_O_14_	577.154	269.046 (100)	Coumestrol-rutinoside	25

**Table 6:** Concentrations of the identified flavonoids in citrus peel extracts.

**Compound**	**Concentration (mg RE/kg)**		
	**Rodriguan grapefruit**	**Mauritian pamplemousses**	**Rodriguan lime**
Naringenin	1200.1	947.8	NI
Hesperidin	NI	NI	661.6
Kaempferol-7-*O*-alpha-l-rhamnoside	NI	NI	11.54
Apigenin 7-*O*-neohesperidoside	72.05	NI	9.03
Apigenin 7-*O*-neohesperidoside isomer	NI	194.9	NI
Apigenin-rutinoside	1481.7	1221.0	NI
6″-*O*-Malonyl naringin	385.7	388.9	NI
Apigenin 7-(6″-malonyl neohesperidoside)	32.6	39.6	NI
Apigenin 7-*O*-(2G-rhamnosyl) gentiobioside	30.9	74.4	NI
Camellianin A (apigenin 7-*O*-glucoside 4′-acetate)	63.8	54.4	NI
Vitexin 2″-*O*-rhamnoside 6″-acetate	497.4	449.0	NI
Diosmin (diosmetin 7-*O*-rutinoside)	828.0	NI	NI
Icariside II	228.9	197.4	393.81
Total flavonoids	4821.1	3178.6	1076.0

Abbreviations: NI: not identified, RE: rutin equivalents.

**Table 7:** Concentrations of identified coumarins in citrus peel extracts.

**Compound**	**Concentration (mg CE/kg)**		
	**Rodriguan grapefruit**	**Mauritian pamplemousses**	**Rodriguan lime**
Meranzin hydrate	722.23	250.0	NI
9-Hydroxy-4-methoxypsoralen 9-glucoside	NI	NI	35.1
Auraptenol	4853.8	2900.6	NI
Oxypeucedanin	159.9	45.5	NI
7-Methoxycoumarin	NI	NI	127.0
Limettin	NI	NI	1372.0
Isopimpinellin	NI	NI	1731.5
Meranzin	5317.3	3287.4	NI
Isomeranzin	2129.6	827.4	NI
Umbelliferone derivative	234.7	162.4	NI
Bergamottin derivative	34.7	701.7	NI
5-Geranyloxy-8-methoxypsoralen	NI	NI	386.5
Bergamottin	NI	46.6	877.7
7-Geranyloxy-6-methoxycoumarin	NI	NI	1616.5
Coumestrol-rutinoside	183.5	404.0	NI
Total coumarins	13476	8626	6146

Abbreviations: CE: coumarin equivalents, NI: not identified.

## Data Availability

Data is available on request from the authors.
